# Clinical Evaluation of *Ex Vivo* Expanded MUC1-Specific Peripheral Blood T Cells for Adoptive Immunotherapy in Relapsed/Refractory Multiple Myeloma

**DOI:** 10.1158/2767-9764.CRC-25-0713

**Published:** 2026-07-07

**Authors:** Erin W. Meermeier, Dara S. Missan, Latha B. Pathangey, Caleb K. Stein, Gabrielle A. Reckard, Susie A. Darvish, Gregory J. Ahmann, Jill Adamski, Rafael Fonseca, Sandra J. Gendler, Michael P. Gustafson, P. Leif Bergsagel

**Affiliations:** 1 Department of Immunology, Mayo Clinic, Scottsdale, Arizona.; 2 Center for Regenerative Biotherapeutics, Mayo Clinic, Phoenix, Arizona.; 3 Division of Hematology and Oncology, Mayo Clinic, Scottsdale, Arizona.; 4 Department of Laboratory Medicine and Pathology, Mayo Clinic, Phoenix, Arizona.; 5 Department of Biochemistry and Molecular Biology, Mayo Clinic, Scottsdale, Arizona.

## Abstract

**Purpose:**

Naturally occurring tumor-specific T cells derived from peripheral blood (PB) offer a clinically accessible source for adoptive immunotherapy. However, the expansion of these T cells from patients with cancer remains a challenge. We hypothesized that mimicking innate immune activation could optimally stimulate antigen-driven T-cell expansion *in vitro*, unlocking the therapeutic potential of PB-derived T cells.

**Patients and Methods:**

We previously developed an *ex vivo* culture system that selectively expands tumor antigen–activated T cells from PB mononuclear cells (PBMC), generating multiclonal effector and central memory T cells. In this hypothesis-generating study, we evaluated the therapeutic potential of MUC1-activated T cells in patients with relapsed and/or refractory multiple myeloma (r/rMM) in a phase I clinical trial (NCT05411497). MUC1 is an oncoprotein overexpressed in r/rMM. We translated our small-scale culture into GMP-compliant, large-scale manufacturing, which achieved expansion of T cells from heavily pretreated patients’ PBMCs without exhaustion. Five patients were treated with escalating doses of up to 1 × 10^10^ T cells.

**Results:**

Although the cell infusions were well tolerated, no objective responses occurred. One patient, who received the highest dose, has had stable disease for 2 years after infusion. This patient exhibited transient dermatitis with localized MUC1 and CD3 staining, as potential evidence of on-target, off-tumor T-cell reactivity, perhaps contributing to disease stabilization. T-cell receptor sequencing revealed the T-cell product in four of the patients’ blood, which correlated with the product’s degree of polyfunctionality and MUC1 reactivity.

**Conclusions:**

These findings demonstrate the feasibility, safety, and biological activity of PB-derived, MUC1-specific T cells as adoptive immunotherapy.

**Significance:**

Expanding tumor antigen–specific T cells for clinical use from the blood of heavily pretreated patients with cancer remains a significant hurdle. We scale up a novel culture method that integrates innate immune signals to stimulate T-cell expansion and demonstrate safety, feasibility, and a case of long-term disease stabilization upon treatment of patients with multiple myeloma in a phase I clinical trial.

## Introduction

Chimeric antigen receptor (CAR) T-cell therapies have transformed the treatment of patients with relapsed and/or refractory multiple myeloma (r/rMM); however, many patients fail to achieve durable responses (1). Expanded natural tumor-reactive T cells may offer advantages over CAR T-cell therapy by leveraging the body’s natural antigen recognition to target a broader range of tumor antigens without genetic modification, resulting in lower toxicity, faster manufacturing, and greater potential for limiting antigen escape.

We recently established an *ex vivo* T-cell culture system that selectively expands tumor antigen-reactive T cells from peripheral blood (PB) mononuclear cells (PBMC), a readily available source of T cells and antigen-presenting cells (APC; refs. 2, 3). Activation of T cells with tumor-specific long peptides presented by APCs induces a multiclonal, antigen-specific response, generating CD4^+^ and CD8^+^ effector and central memory T cells that produce interferon-gamma (IFNγ), granzyme B, and perforin upon secondary restimulation. The long cancer-derived peptides (32–39 amino acids) cover class II and class I epitopes of the most common alleles found in diverse populations and alleviate the need for restriction to specific HLA alleles. Following culture for 19 days, therapeutic numbers of antigen-reactive T cells are harvested, ready for infusion into patients. Our previous data demonstrate that T cells reactive to the MUC1 peptides used in this trial are present at low levels in healthy donors and patients with r/rMM (4). Moreover, this *ex vivo* culture system facilitates the expansion of T cells capable of overcoming several dysfunctions commonly observed in multiple myeloma (4).

MUC1 is a known oncoprotein that promotes resistance to apoptosis and immune escape and is well characterized for its immunogenicity and tumor expression (5, 6). Studies have shown that targeting MUC1 results in multiple myeloma cell death in both *in vitro* and *in vivo* models (7, 8). MUC1 is also being targeted clinically for treating multiple myeloma and acute myeloid leukemia through trials investigating ImMucin (9) and the MUC1-C terminal inhibitor GO-203 with decitabine (NCT02204085; ref. 10), respectively.

As our previous work demonstrated that our novel culture system could drive the expansion of antigen-activated T cells, we desired to test the therapeutic potential of MUC1-activated T cells in a population of patients with r/rMM. As such, our intent was to faithfully reproduce the small-scale culture system into a large-scale system suitable for manufacturing for patients in a clinical trial. This phase I study (NCT05411497) was designed to determine the maximum tolerated dose (MTD) of a MUC1-activated T-cell infusion in patients. The primary objectives of this trial were to determine the toxicity, feasibility, biological activity, and success rate of in-house manufacturing and administration of MUC1-activated T cells in patients with r/rMM.

## Patients and Methods

### Study population and design

The dose-escalation study was approved by the Mayo Clinic Cancer Center Institutional Review Board. Eligible patients included those with r/rMM and Eastern Cooperative Oncology Group (ECOG) status 0 to 1, MUC1 expression in multiple myeloma tumor cells, and measurable disease. Progression by IMWG criteria was not required for trial eligibility. Written informed consent was obtained in accordance with the Declaration of Helsinki and the International Conference on Harmonisation Guidelines for Good Clinical Practice. Each patient underwent leukapheresis collection for a mononuclear cell apheresis [MNC(A)] product, followed by lymphodepletion (LD) chemotherapy [cyclophosphamide (CTX) or bendamustine] on days −5, −4, and −3. MUC1-activated T cells were delivered IV on day 0, and after completion, patients were followed up on days 1 to 3, 7, and 28 and every 90 days. Three patients received escalating suboptimal doses of T cells, whereas the final two patients received the highest and optimal dose. Clinical outcomes measured were, primarily, the incidence of adverse events and, secondarily, clinical response, progression-free survival, and overall survival. Other outcomes monitored were the pharmacodynamics of the immunophenotype of the adoptive T cells, T-cell receptor (TCR) tracking, blood immune profiling, and MUC1 expression. For the analysis of MUC1 expression in bulk RNA sequencing, we examined the CoMMpass interim analysis 22 release clinical data and RNA expression (Salmon TPM with immunoglobulin transcripts removed; ref. 11). Patients were enrolled in the CoMMpass study (NCT01454297), sponsored by the MMRF in accordance with the Declaration of Helsinki. Only baseline bone marrow (BM) samples from patients below 75 years of age were used for analyses (*n* = 653). All analyses were performed using R statistical software with two-sided *t* tests and the Wald test for Cox proportional hazards.

### Cell product manufacturing and characterization

MNC(A) products were sampled for sterility, complete blood count, and flow cytometry prior to processing the blood product. Cells were washed with phosphate-buffered saline (PBS) and were frozen using CS-10, a preformulated cryopreservation medium with 10% dimethyl sulfoxide in vials at 1 × 10^8^ cells per cryovial and were cryogenically stored until thawed for *ex vivo* culture. MUC1-activated T cells were generated at the Human Cellular Therapy Laboratory (HCTL; Mayo Clinic Phoenix campus) for 19 days. PBMCs were thawed and washed with 1× PBS on day 0 and plated at 1.5 × 10^6^ cells/cm^2^ in 100M G-Rex vessels. The patient dose level determined how many G-Rex vessels were plated. For dose levels 1 and 2, a single G-Rex 100M vessel was used. For dose level 3, three G-Rex 100M vessels were seeded. For dose level 4, 12 G-Rex 100M vessels were seeded. On day 1, cells were treated with a MUC1 peptide cocktail, defined in the following methods paragraph, followed by R848 (6 μg/mL) and then LPS (1 ng/mL). Vessels were incubated overnight. On day 2, cells were washed with PBS and placed back in their respective vessels. The vessels were filled with complete AIM V media, including IL7 (50 μg/mL), GlutaMAX (1×), and heat-inactivated human AB serum (2%), and incubated until day 19. On the final day of culture, cells were washed with 2% human serum albumin (HSA) in Plasma-Lyte A. The final formulation of the product administered to the patients was the cell dose in 2% HSA in Plasma-Lyte A. Flow Cytometry (Release Testing): Sample preparation for flow cytometry was adapted from previously designed assays (12). Briefly, cells from the final product were stained with CD45-Krome Orange, CD3-Allophycocyanin (APC), CD4-Alexa-Fluor 700, CD8-PacBlue, and 7-Aminoactinomycin D for 15 minutes. Cells were then lysed for 10 minutes using VersaLyse. Flow-count fluorospheres were added to the samples to quantitate absolute cell numbers. Stained samples were run on the CytoFLEX (Beckman Coulter) and analyzed using Kaluza. Endotoxin and Mycoplasma (Release Testing): Endotoxin testing was performed using the EndoSafe PTS system following the manufacturer’s procedures. Mycoplasma testing was performed using the “QC sample prep kit” for DNA extraction and the MycoTOOL Mycoplasma Real-Time PCR kit from Roche CustomBiotech.

### MUC1 peptide cocktail

The predicted immunologic “hot spots” for the MUC1 protein proved to be within the nonglycosylated SEA protein domain (sperm protein, enterokinase, and agrin; refs. 2, 3). The MUC1-activating cocktail, synthesized by GeneMed, consisted of SEA1 (32mer; SPQLSTGVSFFFLSFHISNLQFNSSLEDPSTD-amide), SEA2 (39mer; STDYYQELQRDISEMFLQIYKQGGFLGLSNIKFRPGSVV-amide), and SEA3 (32mer; DVETQFNQYKTEAASRYNLTISDVSVSDVPFP-amide).

### Immune profiling of cell products

Several panels were used for flow cytometry; antibody details are listed in Supplementary Table S1: general immune phenotyping panel (CD3, CD4, CD8, CD56, CD33, CD19, CD15, CD14), T-cell subsets panel (CD3, CD4, CD8, CD45RA, CD45RO, CCR7), costimulatory/checkpoint inhibitory panel (CD3, CD4, CD8, CD28, PD-1, PD-L1, TIM-3, LAG-3), regulatory T-cell (Treg) panel (CD3, CD4, CD8, CD25, IL7Ra, FoxP3, Helios), and intracellular cytokine staining panel (CD3, CD4, CD8, IFNγ).

On day 19 (after MUC1 activation), a small aliquot of T cells (5–10 mL) in its culture media was transported on ice to the research lab at the Mayo Clinic Scottsdale campus for evaluating its phenotypic expressions and IFNγ specificity. Frozen vials of PBMCs were also obtained from HCTL on dry ice for day 0 evaluations and for restimulation intracellular cytokine (ICC) assay. To evaluate cell phenotype, PBMCs (day 0) and T cells (day 19 after MUC1 activation) were washed with Ca^2+^/Mg^2+^-free PBS (Gibco/Thermo Fisher Scientific, #10010) and stained for 30 minutes at room temperature with Live/Dead UV Blue stain (Life Technologies, #L23105) at a 1:1,000 dilution in PBS for assessing viability. Cells were then washed with FACS buffer [Ca^2+^/Mg^2+^-free PBS containing 1% heat-inactivated fetal bovine serum (Sigma Aldrich, #F2442) and 0.02% sodium azide (Sigma-Aldrich, #S-8032)]. After washing, cells were exposed to Fc receptor block with 50 mg of unconjugated human IgG (Sigma-Aldrich, #I4506) and stained for surface proteins with fluorochrome-conjugated antibodies. For analyzing intracellular proteins, the cells were then subjected to fixation and permeabilization according to the manufacturer’s guidelines (eBioscience, #00-5123-43, #00-5223-56, and #00-8333-56; BD Biosciences, #51-2090KZ, #51-2091KZ), followed by staining for the intracellular proteins. Appropriate isotype control antibodies were used. Flow cytometry data were acquired on Fortessa (BD Bioscience). First, viable cells were gated based on the absence of UV Blue stain. Dot plots showing each cell surface expression were gated on live CD45^+^ cells, and the % frequency of each cell phenotype was analyzed using FACSDIVA software. Graphs were prepared using GraphPad Prism 10 software.

### Restimulation intracellular cytokine assay

A second stimulation of harvested T cells on day 19 was performed to evaluate IFNg specificity. For this, a fresh vial of PBMCs was thawed on day 17, washed, and resuspended in AIM V media (Gibco, #0870112-DK) with 0.5% GemCell plus xeno-free human AB serum (Gemini Bioproducts, #100-912) and amphotericin B (125 ng/mL; Lonza, #17-836E) at a density of 6 × 10^6^ cells/mL. One milliliter of cell suspension was added per well in a 24-well cluster plate (Costar Corning/Sigma-Aldrich, #3524) and incubated at 37°C in a tissue culture incubator (Thermo Fisher Scientific, HERAcell 150) with 5% CO_2_ and humidity. On day 18, each MUC1 (SEA1, 2, 3) peptide and an irrelevant MUC1-negative peptide was added singly to each well at a concentration of 50 μg/mL. On day 19, MUC1-expanded T cells were resuspended at a density of 4 × 10^6^ cells/mL, and each SEA peptide-pulsed, irrelevant (negative) peptide-pulsed, or no peptide (unpulsed) PBMCs were washed and resuspended at a density of 2 × 10^6^ cells/mL in AIM V media with 2% GemCell, plus xeno-free human AB serum and Amphotericin B (125 ng/mL). Restimulation was performed in a round-bottom 96-well cluster plate (Costar Corning/Sigma-Aldrich, #3799) with a ratio of 2 T cells/1 restimulatory PBMC (100 μL of 4 × 10^6^ cells/mL T cells plus 100 μL of 2 × 10^6^ cells/mL restimulatory PBMCs) for 18 hours at 37°C in a tissue culture incubator. For ICC, monensin (GolgiStop, BD Biosciences, #554724) was added as per the manufacturer’s instructions after 4 hours to block the export of endogenously produced cytokines. The cells were then surface-stained for CD3 BV650 (BioLegend, #317324), CD4 BV510 (BD Horizon, #582970), and CD8 APC eFluor 780 (eBioscience, #47-0088-42), followed by intracellular staining for IFNγ (eBioscience, #48-7319-42).

### Single-cell secretome analysis

PBMCs or day 19 antigen-activated T cells were thawed in a 37°C water bath and then washed with either RPMI media (PBMCs) or AIM V media with IL7 and human AB serum (day 19 T cells). Cells were then resuspended at 1 × 10^6^ cells/mL media (PBMCs in RPMI with IL2, or day 19 T cells with AIM V with IL7 and human AB serum). Cells were incubated at 37°C with 5% CO_2_ overnight. The following day, cells were collected and centrifuged for 10 minutes at 300 × *g* at room temperature. Cells were resuspended in the respective media and counted using trypan blue staining. The cells were then centrifuged for another 10 minutes at 300 × *g* at room temperature. If cells were <70% viable, a Ficoll dead cell removal was performed. CD8^+^ cells, followed by CD4^+^ cell selections, were performed serially on the Miltenyi AutoMACS following the manufacturer’s procedures. The positive fractions were counted and then centrifuged for 10 minutes at 300 × *g* at room temperature. Cells were then resuspended in complete media (RPMI for PBMCs, AIM V for day 19 T cells) at a concentration of 1 × 10^6^ cells/mL, and 100 μL per well was plated in a 96-well plate. The remaining cells were then centrifuged at 300 × *g* for 10 minutes at room temperature and resuspended at a concentration of 1 × 10^6^ cells/mL in media containing 5 μg/mL anti-CD28 antibody and then plated into wells coated with anti-CD3. The plate was then incubated at 37°C, 5% CO_2_ for 24 hours. After incubation/stimulation, cells were harvested separately from the plate (unstimulated vs. stimulated, CD4^+^ vs. CD8^+^) and stained with the membrane stain provided with the IsoLight Single-Cell Secretome Adaptive Immune Human kit using the manufacturer’s instructions. After staining, cells were counted and centrifuged at 300 × *g* for 10 minutes at room temperature and then resuspended in either RPMI or AIM V (without IL-7) at a concentration of 1 × 10^6^ cells/mL. Cells were then loaded onto the IsoLight Single-Cell Secretome Adaptive Immune–Human chips, and the assay was run. Data from the runs were analyzed using IsoSpeak software.

### MUC1 staining by flow cytometry

Samples of the patient’s BM aspirate were ACK-lysed and Fc receptors blocked with Trustain FcX (BioLegend, #422301) diluted in FACS buffer. Then cells were stained with the following antibody panel targeting CD38, CD138, CD3, CD45RA, CD19, viability, and MUC1 (clone HMPV; BD Biosciences) or isotype control, washed, and acquired on a Cytoflex LX (Beckman Coulter). Antibody information is listed in Supplementary Table S1.

### MUC1 staining of skin lesions

Immunofluorescence staining was done on 4-micron cut formalin-fixed, paraffin-embedded tissue sections. Slides were deparaffinized through a series of xylenes and alcohols and rehydrated in water. Heat-induced antigen retrieval was done with antigen retrieval solution from Dako (cat. #S1699) in a vegetable steamer for 30 minutes. Slides were then rinsed in water, permeabilized in 0.3% Triton X-100 (Sigma, #T9284) in TBS buffer for 45 minutes, blocked with Background Sniper (Biocare, #BS966H) for 10 minutes, and incubated first in anti-human CD3 (Abcam, #ab52959) and anti-human BC2 (MUC1) overnight at 4°C and then in fluorescently labeled secondary antibodies for 1 hour at room temperature. Primary and secondary antibodies were washed 3 times for 5 minutes in 0.03% Triton X-100 in TBS buffer. The slides were coverslipped in VectaShield mounting media containing DAPI (Vecta Labs, #H-1200). Images were acquired on a Keyence BZ-X800 Microscope.

### TCR sequencing and analysis

The PB for TCR analysis was drawn in vacutainers containing ACD, and MNC was prepared following the standard Ficoll protocol. DNA was extracted using the Puregene kit and quantified with the Qubit. TCR beta (TCRβ; *TRB*) sequencing was performed using the immunoSEQ Assay (Adaptive Biotechnologies), which uses multiplex PCR amplification of the CDR3 region of the TCRβ chain from genomic DNA. High-throughput sequencing was performed, and data were analyzed using the immunoSEQ Analyzer platform to quantify unique clonotypes and determine clonality, Simpson clonality diversity metrics, and the relative abundance of T-cell clones. The track rearrangement tool was used to identify and quantify TCRβ rearrangements present in the cultured product and shared at any time point in PB samples after infusion. We used the NIAID-funded Immune Epitope Database tool TCRMatch (13) to screen for known antigen reactivity of the most expanded TCRβ (above a detectable frequency of 1 × 10^−6^) from patients’ products and blood.

## Results

### Clinical trial and patient demographics

**Figure 1.**
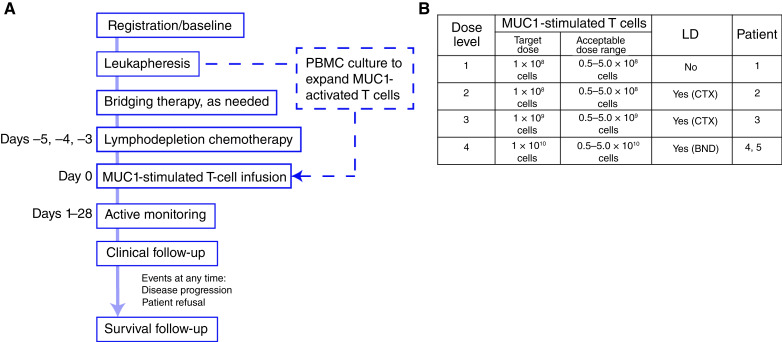
Overview of study design. **A,** Timeline of a phase I clinical trial that assessed the safety and feasibility of *ex vivo* expansion and subsequent infusion of MUC1-stimulated autologous T cells for patients with r/rMM. **B,** Escalating dose levels of MUC1-stimulated autologous T cells and preconditioning LD by patient number

We performed a phase I study in patients with r/rMM to determine the tolerability, feasibility, and biological activity of an autologous MUC1-activated T-cell therapy. Eligible patients included those with r/rMM and ECOG status 0 to 1, MUC1 expression in multiple myeloma tumor cells, and measurable disease. Progression by IMWG criteria was not required for trial eligibility. As toxicity was not expected in the first dose levels, an accelerated titration design was used to identify the MTD level in this patient population. This design allowed more rapid dose escalation if moderate or dose-limiting toxicity (DLT) was not observed and thus limited the number of patients treated at low and potentially subtherapeutic dose levels. If moderate toxicity (a lower tolerability threshold than DLT) was observed in two patients or DLT in one patient, the design would revert to a standard cohorts-of-3 rule-based design. Figure 1 outlines the clinical trial schema. As a phase I study, the primary objective of this trial was to determine the toxicity, feasibility, and success rate of in-house manufacturing and administration of MUC1-activated T cells in patients with multiple myeloma. Five patients were consented and enrolled in the study (no consented patients failed the screening; Supplementary Tables S2 and S3). The patients’ median age was 71 years, and all had received three or more prior lines of therapy.

### MUC1 is a relevant target in multiple myeloma

**Figure 2.**
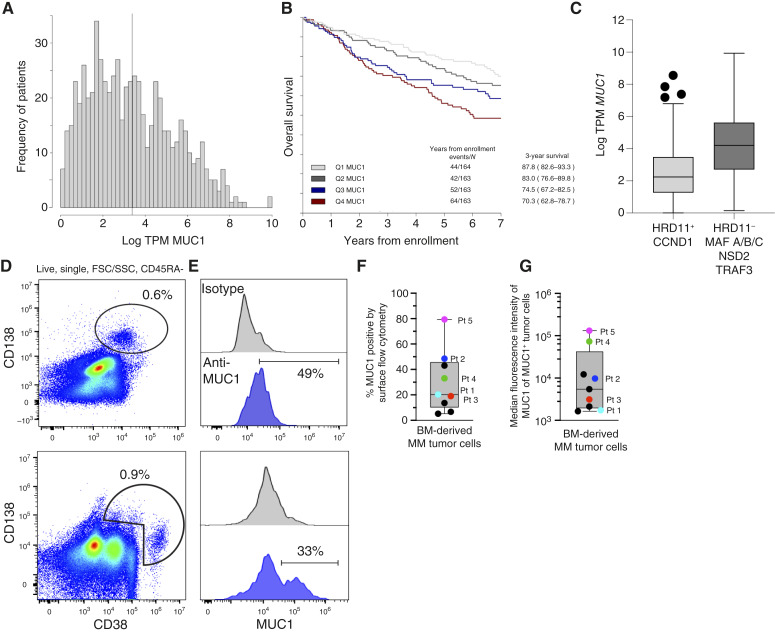
MUC1 is expressed on multiple myeloma tumor cells in a cohort of newly diagnosed patients and in patients at the time of trial enrollment. **A,** Box plot showing the distribution of patient baseline BM samples expressing MUC1 at a range of transcript per million (TPM) reads log values, *n* = 653. The vertical line indicates the optimal binary high and low expression split at the 55th percentile. **B,** Overall survival in a Cox proportional hazards model by quartile of MUC1 TPM expression, with the 3-year survival and (range, years). **C,** Box and whisker plots show the distribution of MUC1 expression with lines at the median and a box around the IQR. Cases were divided into two groups of disease subtypes classified by CCND2 affinity (left, low; right, high), *n* = 653. **D,** Flow cytometry gating of multiple myeloma tumor cells, two examples: patient 2 (top) and patient 4 (bottom). Cells were identified as live, single cells that did not express CD45RA with low to high expression of CD38 and CD138. **E,** Multiple myeloma tumor cells were assessed for staining with the anti-MUC1 antibody above the appropriate isotype control. The gate shows the positive fraction above the isotype. **F,** Box plot summary of the percentage of multiple myeloma tumor cells, by patient, that were MUC1^+^ by flow cytometry. **G,** Box plot of the median fluorescence intensity of MUC1 staining of the multiple myeloma tumor cells identified as MUC1^+^ in **B** and **C**. Patients enrolled in the trial are indicated as different colored dots. Black dots represent other samples of patients with r/rMM screened for MUC1 expression.

We previously found that MUC1 is upregulated in multiple myeloma (as well as other cancers; refs. 3, 5). In a broader context, analysis of 653 baseline multiple myeloma samples (from patients aged <75 years) from the CoMMpass dataset demonstrated widespread and heterogeneous expression of MUC1 at the RNA level (Fig. 2A). Further analysis of these data revealed that MUC1 expression levels were moderately associated with overall survival in this patient cohort, log-rank *P* value 0.001817 (Fig. 2B). We also observed *MUC1* expression to be highly correlated with *CCND2* expression (Supplementary Table S4), a feature known to distinguish primary multiple myeloma disease by genetic subtypes (14). We then split baseline cases according to the *CCND2* affinity of their primary subtype and observed *MUC1* expression to be enriched in subtypes with CCND2 affinity: MAF A/B/C with immunoglobulin heavy chain (IGH) translocations t(8;14), t(14;20), and t(14;16), respectively; NSD2 with IGH translocation t(4;14); hyperdiploid cases without trisomy of chromosome 11 at CCND1; and a remaining subset of CCND2-expressing cases lacking an IGH translocation or hyperdiploid features characterized instead by monosomies of 13q and 14q with common bi-allelic alteration of TRAF3. Alternatively, hyperdiploid multiple myeloma with trisomy of chromosome 11 at CCND1 and cases with IGH translocations with CCND1 [t(11;14)] had significantly lower expression of MUC1 (Fig. 2C).

To validate MUC1 protein presence for trial enrollment and understand its range of expression in tumor cells of our target patient population, we used surface flow cytometry of whole BM aspirates of each patient at enrollment and additional nonenrolled patients. BM plasma cells were identified as double positive for CD38 and CD138 (Fig. 2D, two examples). MUC1 surface expression was quantified as staining above the applicable isotype control (Fig. 2E, two examples). The frequency of tumor cells expressing MUC1 and the density of MUC1 expression on MUC1^+^ tumor cells are summarized by each patient sample (*n* = 9; Fig. 2F and G). All patient samples contained MUC1^+^ tumor cells, which ranged from 5% to 79% of the tumor cells. Of the patients enrolled in the trial, the range in tumor cells expressing MUC1 was 18% to 79%. We observed a wide range in the intensity/density of MUC1 expression, over 2 logs of range in MFI. Tumor cells from all samples tested positive for heterogeneous levels of MUC1 expression, confirming its use as a multiple myeloma target in this patient population.

### Phenotypic characterization of the MUC1-activated T-cell product

**Figure 3.**
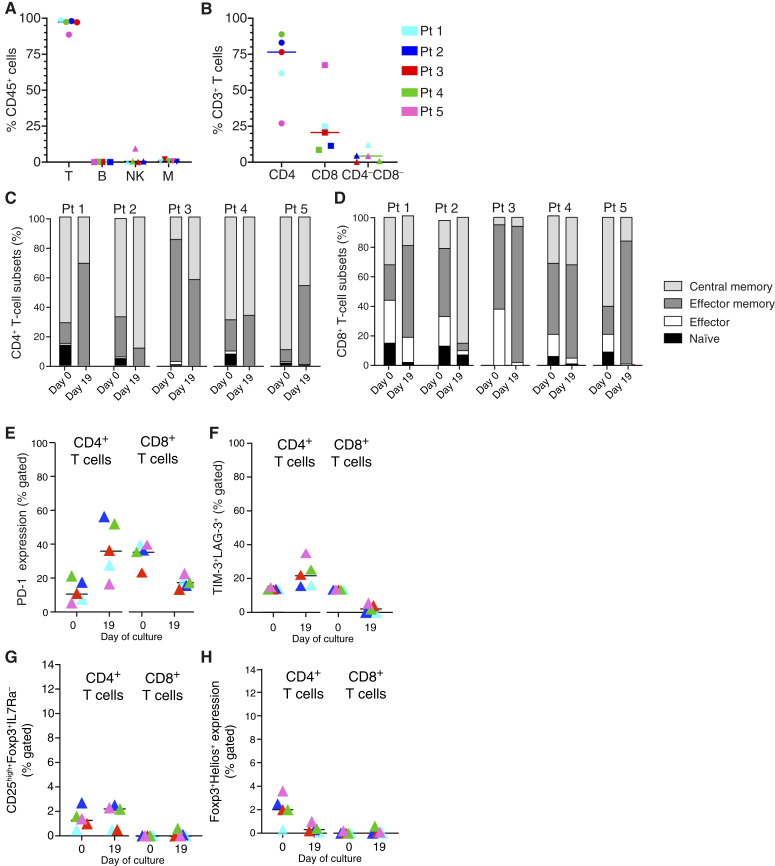
Natural *ex vivo* culture system expands T cells enriched for moderately activated, nonexhausted memory subtypes. **A,** Flow cytometry analysis of cell lineage at the end of culture (day 19). CD45^+^ cells were gated into T cells (CD3^+^), B cells (CD19^+^), NK cells (CD56^+^), and myeloid (M; CD14^+^/CD15^+^/CD33^+^) cells. Each color indicates an individual patient, *n* = 5. **B,** T cells were further gated into CD4^+^ and CD8^+^ subsets, and memory subsets (naïve CD45RA^+^CCR7^+^, effector CD45RA^+^CCR7^−^, effector memory CD45RO^+^CCR7^−^, and central memory CD45RO^+^CCR7^+^) on (**C**) CD4^+^ and (**D**) CD8^+^ T cells at the beginning (day 0) and end (day 19) of culture. Frequency of CD4^+^ or CD8^+^ T cells expressing markers associated with activation and exhaustion, (**E**) PD-1^+^, (**F**) TIM-3^+^LAG-3^+^, or identified as Tregs (**G** and **H**) using two gating strategies (CD25^+^FoxP3^+^IL7Ra^+^ or FoxP3^+^Helios^+^). Each color indicates an individual patient.

Patient PBMCs were obtained from nonmobilized leukapheresis products. We were successful in generating MUC1-activated T-cell products at the appropriate dose in all the patients starting from frozen/thawed PBMCs. The 19-day culture yielded an average purity of 96% T cells, with a minimum of 88.6% (Fig. 3A). CD4^+^ T cells were the predominant expanded T-cell subset, comprising a mean of 67.4%, with CD8^+^ T cells at 26.6% (Fig. 3B).

One patient’s (patient 5) culture behaved very differently, in which 67.5% of the T cells were CD8^+^ T cells and only 27% were CD4^+^ T cells. The T-cell product largely consisted of effector and central memory T-cell subsets. Of the CD4^+^ T cells, 45.2% (range of 12%–69%) were CD45RO^+^CCR7^−^ effector memory T cells, and 54.6% (range of 31%–88%) were CD45RO^+^CCR7^+^ central memory T cells. In CD8^+^ T cells, 61% (range of 5%–92%) were CD45RO^+^CCR7^−^ effector memory T cells, and 31.8% (range of 6%–85%) were CD45RO^+^CCR7^+^ central memory T cells. In most cases, effector and central memory populations were preferentially enriched in the final product compared with the initial population of T cells in the leukapheresis product (Fig. 3C and D). The expanded day 19 T cells did not show high expression of exhaustion markers. Although PD-1 expression did increase on CD4^+^ T cells from 12.1% on day 0 to 37.3% on day 19 (n.s.), PD-1 expression was lower on day 19 CD8^+^ T cells (17.4%) than on day 0 (23.9%; Fig. 3E). T cells expressing other exhaustion markers, TIM-3 and LAG-3, did not increase after the 19-day culture (Fig. 3F). Finally, Tregs were not enriched in the day 19 product as CD4^+^CD25^hi^Foxp3^+^IL7Ra^−^ and CD4^+^Foxp3^+^Helios^+^ Treg populations were not different from the percentages of Tregs in the day 0 apheresis product (Figs. 1, 3G and H).

### Culture process generates potent T cells that target MUC1

**Figure 4.**
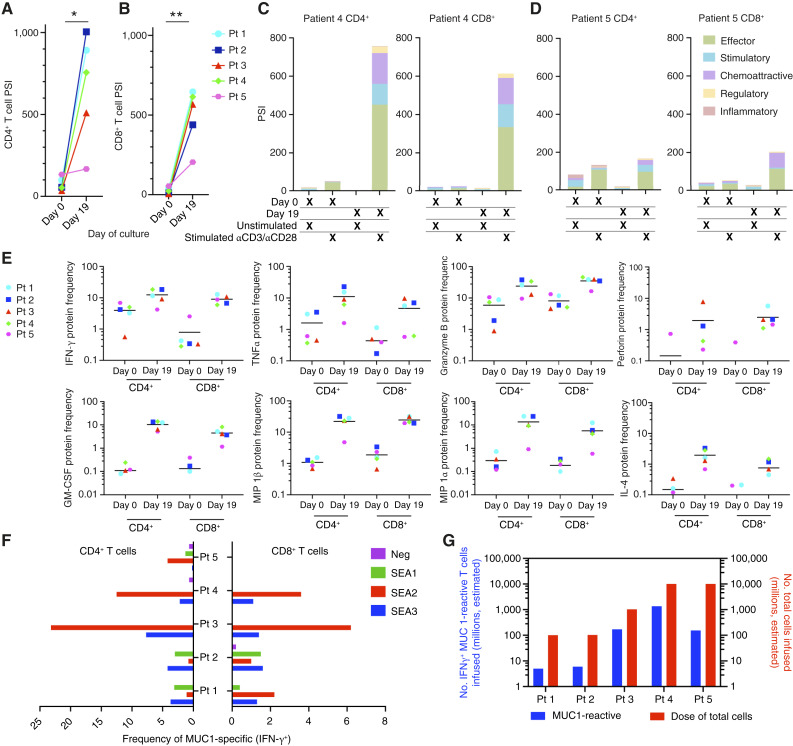
Natural *ex vivo* culture system expands polyfunctional T cells enriched for MUC1 antigen–specific reactivity. **A,** CD4^+^ and (**B**) CD8^+^ PBMC-derived T cells at baseline of culture or day 19, the end of culture, were subjected to single-cell secretome analysis using IsoLight technology. T cells were left unstimulated or stimulated for 24 hours with anti-CD3/CD28. The *y*-axis lists the overall functional potency of the cell population as PSI, which integrates how many cytokines and at what intensity they are secreted. Each color indicates an individual patient. **C,** Patient 4 and (**D**) patient 5 PSI for CD4^+^ and CD8^+^ T cells, in which each color represents the functional grouping of cytokines. Effector: IFNγ, TNFα, and granzyme B; stimulatory: IL2 and IL12; chemoattractive: MIP-1α, MCP-1, and IP-10; regulatory: IL10 and TGF-β; and inflammatory: IL6, IL1β, and TNFα. **E,** Frequency of CD4^+^ or CD8^+^ single T cells producing select cytokines at baseline or the end of culture. Bar indicates the mean frequency. Each color indicates an individual patient. **F,** Frequency of CD4^+^ or CD8^+^ T cells, by patient, that responded to MUC1-derived SEA peptides or an irrelevant peptide negative (neg) control peptide with IFNγ secretion, detected by ICC staining and flow cytometry. **G,** Estimated amount of MUC1-specific T cells (blue) infused per patient based on (**F**) and dose (red). *P* values were determined by an unpaired *T* test: *, *P* < 0.05; **, *P* < 0.01.

To add to our phenotypic characterization of the MUC1-activated T-cell product, we examined the cultured T cells with a focus on their functional responses and antigen specificity. We used single-cell multiplexed proteomics to measure the polyfunctional strength index (PSI) of day 19 cells as compared with the day 0 T cells. Polyfunctional T cells, which produce at least two cytokines, play a crucial role in robust antitumor immune responses, and their activity can be measured using the PSI—a metric combining the proportion of these cells secreting multiple cytokines with the average intensity of the proteins they secrete (15). Samples from day 0 and day 19 CD4^+^ and CD8^+^ T cells were stimulated via CD3/CD28 stimulation or tested unstimulated. Every patient increased in PSI after 19 days of culture for both CD4^+^ and CD8^+^ T cells, ranging from a 1.3-fold to an 18.2-fold increase in CD4^+^ T cells and a 3.7-fold to a 94.7-fold increase in CD8^+^ T cells (Fig. 4A and B). Specific examples of two different T-cell responses to the culture process are shown in Fig. 4C and D. Day 19 CD4^+^ and CD8^+^ T cells from patient 4 demonstrated substantial improvement in PSI with strong induction of effector cytokine secretion (Fig. 4C). In contrast, day 19 T cells from patient 5 exhibited only modest improvement in PSI with higher baseline (day 0) secretion in unstimulated samples, reflecting high inflammatory and stimulatory cytokines (Fig. 4D and E). Further analysis of populations secreting individual cytokines shows that day 19 cells displayed increased secretion of Th-1 promoting effector cytokines, including IFNγ, TNFα, granzyme B, and Perforin (Fig. 4E). This pattern of increased expression of established surrogate markers of cytotoxic potential mirrors our previous findings using the same culture system; PBMCs of patients with multiple myeloma robustly acquired expression of cytolytic machinery, with nearly all CD8^+^ T cells expressing perforin and granzyme B (3). GM-CSF, MIP-1α, and MIP-1β were other cytokines that were enriched in day 19 T cells. Although the proportion of IL4-secreting T cells did increase on day 19, their overall abundance in the product is quite low.

To measure antigen specificity, day 19 T cells were restimulated with day 0 PBMCs. Patients 1 and 2 exhibited reactivity to all three MUC1 (SEA) peptides, patients 3 and 4 to just SEA2 and SEA3, and patient 5 only had reactivity in the CD4^+^ subset to SEA1 and SEA2 (Fig. 4F). Finally, Fig. 4G illustrates the actual dose of MUC1-reactive T cells for each patient compared with their total dose. Patient 4 received the highest dose of MUC1-reactive T cells, and although patient 5 received the same total cell dose as patient 4 (1 × 10^10^ cells), patient 5 only received about one tenth the MUC1-reactive dose as patient 4 (155 million vs. 1,366 million, respectively; Fig. 4G). The phenotypic and functional data suggest that the cultured MUC1-activated T cells yield memory T cells that are not exhausted, have increased capacities to secrete Th-1 promoting cytokines, and are enriched for reactivity to the tumor antigen, MUC1.

### Safety

Cells were infused as a fresh product with LD (patients 2–5) or without (patient 1). Treatments were generally safe as no infusion toxicity, cytokine release syndrome, immune effector cell-associated neurotoxicity syndrome, or severe autoimmunity was observed. In addition, no DLTs were observed. Overall, 16 adverse events were reported, with 5 (31%) grade 3 and 1 (6%) grade 4 events (Supplementary Table S5). All grade ≥3 events except for one (grade 3 hypertension) were related to LD. One case of transient grade 2 dermatitis may have been linked to the study drug and is discussed in more detail below. No other events were linked to the study drug.

### Patient responses

The first three patients received doses that we predicted to be suboptimal, as part of the safety lead-in. The first patient had no LD chemotherapy, whereas the next two patients were treated with CTX for LD. To achieve deeper LD, the final two patients were treated with bendamustine for LD chemotherapy. The dose of T cells infused was increased stepwise (Fig. 1), in which the final two patients also received the ideal dose of 1 × 10^10^. There were no objective responses in terms of M-spike reduction. Disease progression occurred in four patients at 2, 4, 4.5, and 12.2 months, and they subsequently received Ciltacabtagene autoleucel (Cilta-cel) CAR T-cell therapy. These patients remain in MRD-negative complete remission 21 to 38 months after CAR T-cell infusion. In contrast, one patient (patient 4) continues with stable disease at >32 months following the cell infusion with no additional treatment. Although this patient experienced periods of stability on prior therapies, each subsequent line of treatment was associated with progressively shorter durations of disease control. Specifically, this patient progressed within 17 months after switching from pomalidomide–bortezomib–dexamethasone to bortezomib–dexamethasone in 2018 and within 3 months of discontinuing daratumumab–pomalidomide–dexamethasone in 2019. Given this trajectory, sustained disease stability for more than 32 months following discontinuation of ixazomib–pomalidomide–dexamethasone—without maintenance therapy—would not have been expected under the null hypothesis of no treatment effect.

**Figure 5.**
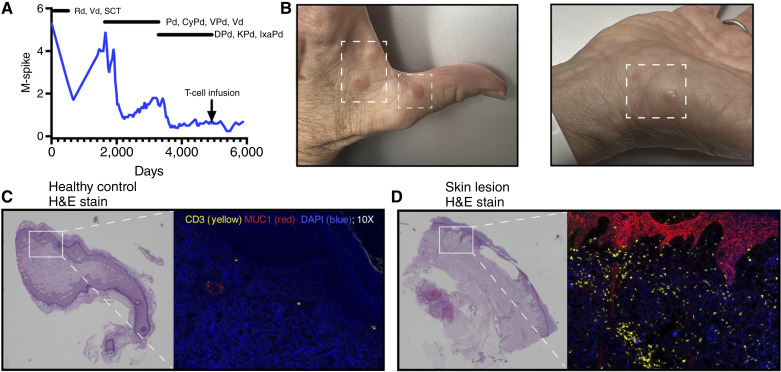
Clinical response to MUC1-activated autologous T cells in a single patient. **A,** M-spike (gamma to albumin ratio) over time in patient 4 treated with MUC1-activated autologous T cells after a series of standard-of-care regimens. D, Darzalex (daratumumab); d, dexamethasone; Ixa, Ninlaro (ixazomib); K, Kyprolis (carfilzomib); P, Pomalyst (pomalidomide); R, Revlimid (lenalidomide); SCT, stem cell transplant; V, Velcade (bortezomib). The arrow indicates the initiation of treatment on trial. **B,** Lesions on the hands of patient 4 (white boxed areas). **C** and** D,** Immunofluorescence staining of lesion or healthy control skin area biopsies of patient 4’s hand. Tissue sections were stained with hematoxylin and eosin (H&E; left) or antibodies specific to CD3 (T cells), MUC1 (antigen), and DAPI and imaged at 10× magnification. White boxes indicate the area of the tissue section that was magnified for fluorescence analysis (right).

Patient 4, who received the highest cell dose and the most polyfunctional T cells, demonstrated the most robust disease stability among the five patients. She had been maintained on continuous pomalidomide-based therapy for the preceding 9 years (Fig. 5A). Following infusion, her C-reactive protein peaked at 13.9 mg/L on day 2. Beginning on day 3, she developed 4 to 9 mm pink, firm, tender dermal nodules with central atrophy and pitting, arranged in annular, clustered, well-circumscribed patterns and localized to both hands and forearms (Fig. 5B). The lesions persisted through day 28, with minimal residual presence through month 3. To determine whether the lesions reflected on-target but off-tumor activity, we examined several lesions by IHC for specific MUC1 staining and CD3 T-cell staining. Although uninvolved skin had no MUC1 reactivity (Fig. 5C), marked expression of MUC1 in the epithelium with extensive lymphocytic infiltrate was evident in the lesions (Fig. 5D).

### TCR sequencing identifies expansion of T-cell product–associated TCRβ *in vivo*

**Figure 6.**
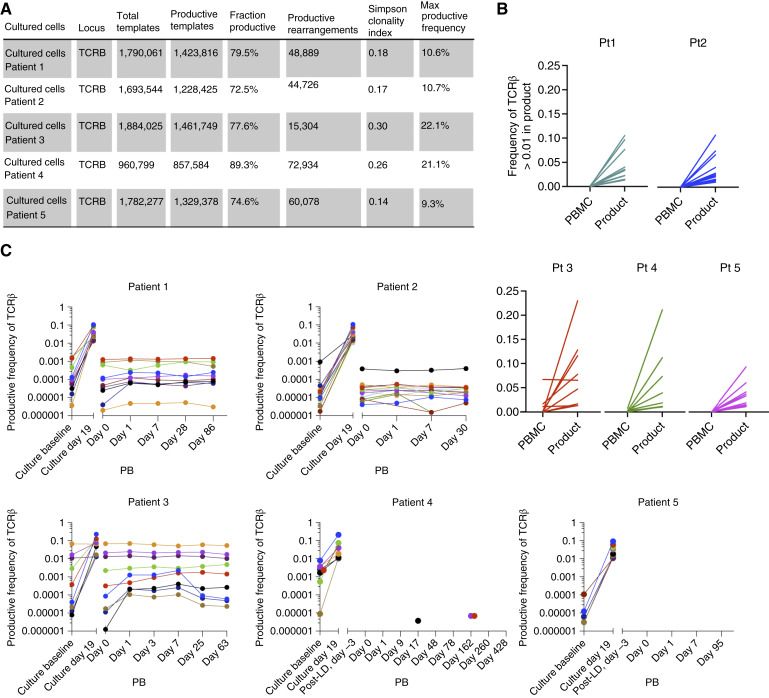
TCR sequencing allows for longitudinal *in vivo* tracing of rearrangements associated with the T-cell product. **A,** Characteristics of sequencing results of the TCRβ repertoire of each patient’s T-cell product. **B,** Frequency of single TCRβ rearrangements in baseline PBMC or day 19 T-cell product by each patient. Each line indicates a single TCR β rearrangement, selected from those that made up 1% or more of the patient’s day 19 T-cell product. **C,** Longitudinal tracking of single TCRβ rearrangements (by color). For patients 1–3 and 5, only the TCRβ present at >1% of the day 19 T-cell product are displayed. For patient 4, all three additional TCRβ present in the day 19 T-cell product and at subsequent time points after infusion are displayed. Culture baseline is the PBMC apheresis product before LD chemotherapy.

To track the MUC1-activated T cells *in vivo*, TCR repertoire analysis was performed using targeted sequencing of the TCRβ (*TRB*) in initial PBMC (prior to LD), cultured day 19 T-cell product, and longitudinal blood samples. Sequencing results for all five patients’ day 19 products are summarized in Fig. 6A. A median and range of 1.78 × 10^6^ and 9.6 × 10^5^ to 1.88 × 10^6^ total DNA templates, respectively, were detected across the day 19 T-cell products. Of these, a median and range of 77.6% and 72.5% to 89.3%, respectively, were predicted to create a productive *TRB* sequence. Ultimately, a median and range of 48,886 and 15,304 to 72,934, respectively, productive rearrangements, or the count of unique functional protein TCRβs, were detected across the five samples. The cultured T-cell product from patients 3 and 4 displayed a higher productive Simpson clonality index, indicating a lower level of diversity of clones and more oligoclonal expansion than the other three patient samples (Fig. 6A and B). We then sought to track *TRB* enriched in the MUC1-activated T cells at different time points after infusion (Fig. 6C). The top enriched *TRB* (more than 1% of productive rearrangements) in patient 1’s product, who received no LD and the lowest dose of T cells, was found at similar frequencies to pretreatment levels at nearly all time point samples. One *TRB* (dark blue line) was ∼11- to 15-fold higher on days 1 to 86 compared with day 0, indicating expansion *in vivo*. Patient 2, who received Cy-based LD and the lowest dose of T cells, showed a similar stability of the products’ top *TRB* before and after treatment, without indication of any expansions. The top *TRB* from patient 3’s (Cy-LD and 10-fold higher T-cell dose) product showed the clearest signs of expansion *in vivo*, with four rearrangements displaying ∼5 to 150-fold expansion over the first week after infusion. These expanded *TRB* then contracted to levels slightly higher than those found at baseline by the end of monitoring on day 63, suggesting a transient expansion of multiple prominent T-cell clones in the product. The remaining enriched *TRB* from this patient’s product were at similar levels before and after treatment at all time points sampled.

As these three patients displayed evidence of inadequate LD, the final two patients were switched to bendamustine for LD. The top *TRB* from patient 4’s and 5’s (both received the highest T-cell dose) products were not found at equivalent levels before and after treatment, a likely indication of the deeper LD. In patient 4, notably, three of the total productive rearrangements were transiently detectable at days 17 and 162, suggesting a low-level presence of the product durably in circulation. Given that this patient developed skin lesions, we queried public databases to see whether the expanded TCRs had known reactivity to antigens from dermal viral infections. Using the top 40 most prevalent TCRβ from this patient’s product, only one of them matched exactly to a TCR with known reactivity (to a SARS-CoV-2 antigen), and none were reactive to dermal viruses such as polyomaviruses. For patient 5, none of the rearrangements present in the product were detectable after infusion, indicating a lack of *in vivo* survival.

In summary, in four of five patients treated with the MUC1-activated T-cell product, the *TRB* most expanded in the product were also detected in the PB after infusion. This occurred in the patient without LD and in patients lymphodepleted with either CTX or bendamustine. In patient 5, where none of the *TRB* of their MUC1-activated T-cell product were detectable after infusion, it is noted that this product also displayed poorer T-cell functional characteristics *in vitro*, such as lower MUC1-antigen specific reactivity. Finally, in three of the five patients (patients 1–3) treated with the MUC1-activated T-cell product, we show evidence of transient *TRB* expansion of the *TRB* associated with their T-cell product, indicating *in vivo* biological activity and stability.

## Discussion

We report results of a hypothesis-generating phase I dose escalation study of MUC1-activated autologous T cells to treat multiple myeloma that had been relapsed or refractory compared with standard therapy. Five patients received a single infusion of autologous T cells that had been cultured using a novel system integrating innate stimuli followed by MUC1 antigen-specific stimulation. The treatment was well tolerated, with only a single adverse event possibly linked to the study drug in one patient (grade 2 dermatitis). All five patients had stable disease for several months after infusion. The autologous T-cell product was detectable, by TRB sequencing, in the blood of four of five patients after infusion, with evidence of expansion in three of five patients. Four patients eventually progressed and received subsequent therapy (all anti-BCMA CAR T cell; Cilta-cel) after an average of 6 months. These four patients achieved a complete response with CAR T-cell therapy and remain in remission. A single patient, patient 4, has had stable disease ongoing for nearly 3 years after infusion without the need for any other interventions. This patient also had evidence of on-target, off-tumor antigen–specific T-cell reactivity. They exhibited transient dermatitis of the hands, which, upon pathologic analysis, had an abundance of MUC1 and CD3 (T-cell) colocalization, indicating T-cell infiltration specifically at sites of high MUC1 protein in the skin, but not at nondiseased skin regions. Although our primary objective of tolerability was met, we acknowledge several limitations, including limited clinical activity, small sample size, heterogeneous MUC1 expression, and suboptimal dosing in early cohorts, which constrain conclusions about efficacy.

Here, we utilized our established sequential culture stimulation approach (2) clinically. This approach first instigates APC activation by exposing PBMC to innate stimuli, GM-CSF plus LPS and R848, to present long MUC1 peptides to CD4^+^ T cells or cross-present to CD8^+^ T cells. Then, IL7 cytokine is added to activate and promote the expansion of antigen-specific memory T cells. In our trial, this approach yielded MUC1 antigen–specific T cells from all five patients’ PBMC tested. Phenotypic characterization of the MUC1-activated T-cell products revealed that all patients’ T-cell cultures were a mixture of central and effector memory T-cell subtypes and were enriched for CD4^+^ T cells. Four of the five patients’ products contained highly polyfunctional T cells, whereas the final patient’s product T cells exhibited lower polyfunctional scores. Similarly, four of the five patients’ products exhibited reactivity to all three MUC1 peptides from both the CD4^+^ and CD8^+^ compartments, with the final patient’s product T cells reacting to a much lower degree. Specifically, this patient’s product had no CD8^+^ T-cell antigen–specific reactivity, despite the majority of the T cells being CD8^+^.

The two patients in this trial who received the highest dose and strongest LD, patients 4 and 5, had disparate outcomes. The functional testing of the expanded T cells provides evidence of product potency. The patient who exhibited extended disease stabilization (patient 4) had T cells with strong PSI, highly reactive to MUC1 activity in both *ex vivo* assays and *in vivo* sequelae. This patient was in the highest dose level group but also received the highest estimated dose of MUC1-reactive T cells based on *in vitro* functional testing (1,366 million vs. 170 and 155 million in patients 3 and 5, respectively). Patient 5 also received the highest dose yet; in contrast, their product exhibited poorer PSI, was less reactive to MUC1, and went on to CAR T-cell therapy within 4 months. Additionally, patient 5 had a more unfavorable prognostic disease subtyping: IgA-λ with a chromosome 1q amplification (four copies); this type of high-risk disease is associated with resistance to therapies and more aggressive disease. Further investigation is needed to determine the underlying factors that contribute to the efficacy of the culture system to expand antigen-specific T cells, but we speculate that a lack of *in vivo* T-cell priming to the MUC1 antigen during the natural history of the patient’s disease or immune aging of the patient’s PBMCs may play a role.

Given the limitations of CAR T-cell therapies, particularly in the context of the safety profile and antigen specificity for solid tumors, further development of adoptive T-cell approaches harnessing native TCR-mediated antigen recognition is both justified and promising. In a previous study, autologous multitumor-associated antigen (mTAA)-specific T cells were tested in patients with high-risk r/rMM, demonstrating safety and promising efficacy (16). Among 21 treated patients, including those with active disease after multiple prior therapies, the infused T cells were well tolerated and associated with longer-than-expected progression-free survival. Objective responses correlated with the presence of functional TAA-reactive T-cell clonotypes derived from the infused product. In this study, CD14^+^ monocyte-derived dendritic cells were generated and pulsed with the multipeptide mix. T cells derived from patient PBMCs were then cocultured and expanded to generate the mTAA-T cells. Although we observed similar patient safety profiles, outcomes, and successful manufacturing as in the study of Lulla and colleagues, there are some significant differences in our study that may help inform the design of future trials using antigen-specific T cells. In the manufacturing of our MUC1 T-cell product, we utilized the existing APCs within the leukapheresis product and used three peptides from one tumor antigen (rather than the multiple antigen approach) for presentation to T cells within the culture. Our culture system yielded primarily CD4^+^ central memory cells, whereas the Lulla study generated a majority of effector memory CD8^+^ T cells. We were able to infuse high quantities of antigen-specific T cells. For patients receiving the highest dose, we manufactured more than 1 × 10^10^ total cells, which represents nearly a 100-fold higher dose than typical CAR T-cell infusions (approximately 1 × 10^6^/kg). We highlight these differences only to emphasize how manufacturing processes, therapeutic design, and implementation may affect the potency of the T-cell product.

A key limitation of this study was the modest number of patients treated, which was due, in part, to the new therapeutic availability of FDA-approved bispecific antibodies and CAR T-cell therapies that affected patient accrual. The emergence of T-cell therapies for multiple myeloma presents a competitive and evolving therapeutic landscape, posing challenges for the further development of adoptive T-cell strategies in this setting. Additionally, targeting a single tumor antigen, MUC1, may have facilitated immune escape mechanisms, potentially limiting therapeutic efficacy. Nonetheless, given our prior work characterizing MUC1 and its role as an immunogenic oncoprotein in multiple myeloma, we considered it important to evaluate its suitability in an adoptive T-cell product. Given the results of this study, we acknowledge that MUC1 may not represent an optimal antigen in multiple myeloma, particularly given interpatient and intratumoral variability in expression. Future studies to target MUC1 on tumor cells may be guided by patient selection based on primary genetic disease subtypes associated with higher MUC1 expression (higher *CCND2* affinity) and technically by quantification of MUC1 molecules per cell, the lack of which was a limitation of our study. Our observed clinical outcomes, potential disease stabilization, are consistent with prior MUC1-based vaccine studies, which highlight the need for improved antigen selection based on tumor types in future T cell-based strategies.

In summary, we successfully manufactured and administered the intended cell dose for each participant without observing any DLTs, underscoring the feasibility and safety of the approach. Our study provides a foundation to expand this natural culture system to generate tumor-reactive autologous T cells targeting other or multiple antigens and for other cancers. Insights gained from this trial have now directly informed the design of a parallel proof-of-concept study in ovarian cancer (NCT06483048), in which MUC1 expression is more homogeneous and robust.

## Supplementary Material

Supplementary Figure 1 Patients Clinical Course

Supplementary Table 1. Flow cytometry reagents

Supplementary Table 2. Patient Demographics

Supplementary Table 3. Representativeness of Study Participants

Supplementary Table 4. MUC1 correlation with other genes

Supplementary Table 5. Adverse events

## Data Availability

All data are available within this article or upon reasonable request to the corresponding author. All other clinical trial data from this study are not publicly available for patient privacy but are available upon reasonable request to the corresponding author. Datasets and protocols are available in the article and from the corresponding author upon reasonable request. The RRIDs of the materials used in this study can be found in Supplementary Table S1. The clinical trial data generated in this study are not publicly available due to privacy concerns but are available from the corresponding author upon reasonable request.
