# Supplementary figures and images for: Clinical Evaluation of Ex Vivo Expanded MUC1-Specific Peripheral Blood T Cells for Adoptive Immunotherapy in Relapsed/Refractory Multiple Myeloma
Source: Cancer Res Commun. 2026 Jul 7;6(7):1592–604. doi: 10.1158/2767-9764.CRC-25-0713 (PMC13341006; doi:10.1158/2767-9764.CRC-25-0713)

Supplemental Figure 1. Patients Clinical Course

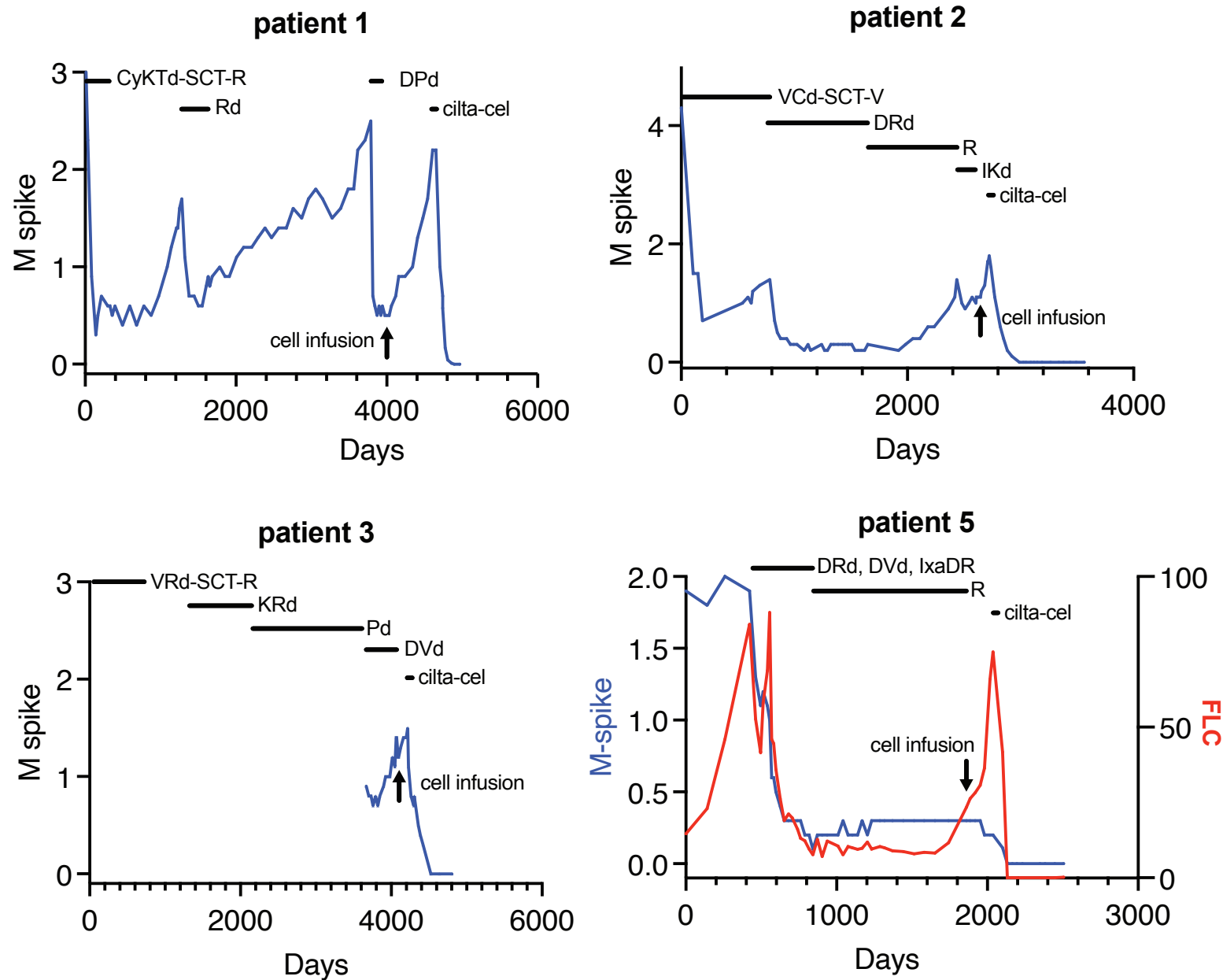

Supplement: Supplementary Figure 1 — Patients Clinical Course [file crc-25-0713_supplementary_figure_1_suppsf1.pdf]
